# Vitamin E discussion forum position paper on the revision of the nomenclature of vitamin E ^☆^

**DOI:** 10.1016/j.freeradbiomed.2023.06.029

**Published:** 2023-07-16

**Authors:** Angelo Azzi, Jeffrey Atkinson, Nesrin Kartal Ozer, Danny Manor, Maria Wallert, Francesco Galli

**Affiliations:** a School of Graduate Biomedical Pharmacology and Drug Development Program, Tufts University, Boston, MA, USA; b Department of Chemistry and Centre for Biotechnology, Brock University, St. Catharines, L2S3A1, Ontario, Canada; c Department of Biochemistry, Faculty of Medicine, Uskudar University, 34662, Uskudar, Istanbul, Turkey; d Department of Nutrition, School of Medicine, Case Western Reserve University, 44106, Cleveland, OH, USA; e Biochemistry and Physiology of Nutrition, Institute of Nutritional Science, Friedrich Schiller University, Jena, Germany; f Human Nutrition and Nutrigenomics Lab, Dept of Pharmaceutical Sciences, University of Perugia, 06122, Perugia, Italy

**Keywords:** vitamin E, RRR-α-tocopherol, tocopherols, tocotrienols, tocomonoenols, AVED, nomenclature, nutrition

## Abstract

This position paper opens a discussion forum of this Journal dedicated to a scientific debate on Vitamin E nomenclature. With this article we provide the scientific and medical communities with what we consider relevant information in favor of revising the nomenclature of vitamin E. To our knowledge, only *RRR*-α-tocopherol has been medically used to protect against a deficiency disease in humans, and therefore, it would be appropriate to restrict the term vitamin to this molecule. The direct demonstration of a vitamin function to other tocochromanols (including other tocopherols, tocotrienols and eventually tocomonoenols), has not yet been scientifically shown. In fact, the medical prescription of a molecule against the deficiency disease only because it has been included in the “Vitamin E family”, but not tested as vitamin E, could lead to ineffective therapy and potentially dangerous consequences for patients.

The idea of this revision launched during the recent 3rd Satellite Symposium on Vitamin E of the 2022 SFRR-Europe meeting, offers a open platform of discussion for the scientists involved in vitamin E research and scientific societies interested to this subject.

## Revision proposal

1. To restrict to *RRR*-α-tocopherol the attribute of vitamin
2. To describe other analogues, including 6-hydroxy-chromanols (i.e. non-α-tocopherols and tocotrienols) and 6-hydroxy-chromenols, by their chemical names until a disease is identified that is prevented by the specific compounds. If such activity is documented, a molecule can be called a bona fide vitamin.
3. Tocochromanols/tocomonoenols with documented vitamin function could be named “vitamin Ex”, where “x” indicates a number, that would be different for the different disease that is prevented. This would be in analogy with the vitamins of the B group, but other definitions can be considered.

A vitamin is defined by the Collins English Dictionary as “Any of a group of substances that are essential, in small quantities, for the normal functioning of metabolism in the body. They cannot usually be synthesized in the body, but they occur naturally in certain foods: insufficient supply of any particular vitamin results in a deficiency disease”. Similarly, the definition provided by Wikipedia is “A vitamin is an organic molecule that is an essential micronutrient that an organism needs in small quantities for the proper functioning of its metabolism”.

Vitamin E deficiency was first reported as a human disease in 1981 [1] and it is known as AVED (Ataxia with Vitamin E Deficiency; OMIM 277460), inherited as an autosomal recessive trait [2-4]. Affected individuals present with peripheral neuropathy and spinocerebellar ataxia. Vitamin E deficiency may also be secondary to other conditions [5], including the impairment of intestinal absorption of lipids and disorders of fat metabolism and bile secretion. These situations may be the consequence of cholestasis, cystic fibrosis, primary biliary cirrhosis and abetalipoproteinemia. Also, obesity [6,7] and fatty liver disease [8-10] induce vitamin E sequestration in fat depots, leading to subclinical conditions of deficiency, and preliminary evidence is suggesting that intestinal dysbiosis may reduce vitamin E bioavailability [11].

AVED patients are treated lifelong with vitamin E preparations that contain *RRR*-α-tocopherol [12]. Such interventions often stop the progression of the disease and sometimes improve neurological symptoms [13,14]. In fact, AVED patients have low *RRR*-α-tocopherol concentrations in plasma and supplementation with alpha-tocopherol produces a normalization of the plasma level and disease remission [15]; no supplementation with other tocopherols can logically increase the plasma level of the missing α-tocopherol since there is clear evidence that the different tocols do not convert into each other. Therefore, the treatment of AVED has only been confirmed with *RRR*-α-tocopherol or mixtures that contain this form, and this is also the case of other vitamin E deficiencies that present with neuromuscular disturbances similar to those associated with AVED (reviewed in Ref. [5]).

There are a number of vitamin E analogues, other than *RRR*-α-tocopherol, present in plants [16], that have been given the attribute of vitamin, without direct evidence of their ability to cure human vitamin E deficiencies [17]. Such an attribute has been the result of hypotheses on the mechanism of action of *RRR*-α-tocopherol in protecting against pathological outcomes.

Firstly, tocopherols including *RRR*-α-tocopherol tested in rat were, with varying efficiency, able to prevent the fetal resorption caused by the deficiency. Humans exhibit different clinical presentations of the disease and no cases of fetal resorption due to vitamin E deficiency have been reported (as a matter of fact, fetal resorption does not occur in humans); therefore, it is our understanding that the extrapolation that a molecule is a vitamin in humans because it is such in rats, is not fitting.

Secondly, the hypothesis has been made that all tocopherols and tocotrienols act by virtue of their antioxidant properties and, consequently, the protection against vitamin E deficiency had to be an antioxidant effect shared by those molecules (reviewed in Ref. [18] and further reviewed in Refs. [19,20]). However, distinct molecular mechanisms for the actions of tocopherols, tocotrienols and tocomonoenols have been described, both *in vitro* and *in vivo* [16,21,22]. Thus, the conclusion that the protection against the deficiency be solely due to an antioxidant effect and that all tocopherols, tocotrienols and tocomonoenols are protective against vitamin E deficiency is not justified.

Third, the close structural similarity between tocopherols and tocotrienols, has frequently led some to suppose that their molecular function must be the same. Such an argument can be potentially dangerous. In fact, in nature a minor difference in a molecule’s structure leads to dramatically different biological activities. Just as an example in the field of vitamins, the anti-scurvy effect of l-ascorbate is not provided by the stereoisomer d-ascorbate; retinoic acid has very different activity profile compared to retinol, and PUFAs have very different biological activities compared to saturated fatty acids, and so on. In the case of vitamin E, Schuelke while revising the literature on AVED for the National Library of Medicine book GeneReviews [12], described the treatment of choice for the disease manifestations in these terms: “It is currently unknown whether affected individuals should be treated with *all*-*rac*-α-tocopherol acetate or with *RRR*-α-tocopherol. It is known that α-Tocopherol Transfer Protein (αTPP) stereoselectively binds and transports 2R-α-tocopherols [23-25]. For some TTPA pathogenic variants, this stereoselective binding capacity is lost and affected individuals cannot discriminate between *RRR*-α-tocopherol and *SRR*-α-tocopherol [4,26]. In this instance, affected individuals would also be able to incorporate non-*2R*-α-tocopherol stereoisomers into their bodies if they were supplemented with *all-rac*-α-tocopherol.”. Based on these aspects, Schuelke concluded that “Since potential adverse effects of the synthetic stereoisomers have not been studied in detail, it seems appropriate to treat with *RRR*-α-tocopherol, despite the higher cost.”. We share these considerations on the importance of giving scientifically based information on AVED treatment underlying that all the tocopherols or tocotrienols are not biologically equivalent to *RRR*-α-tocopherol.

Lastly, we note that evolution has equipped eukaryotes with a powerful and complex biochemical system dedicated to discrimination between the different tocochromanols and tocomonenols; in both animals and humans, these include αTTP that selectively retains *RRR*-α-tocopherol for incorporation into circulating lipoproteins, whereas all the other forms are preferentially excreted or degraded by CYP450-mediated ω-hydroxylation and subsequent β-oxidation-like shortening of the side-chain (reviewed in Ref. [19]). Together, these systems ensure the selective accumulation of *RRR*-α-tocopherol in tissues, and the efficient removal of all other forms. It is only reasonable to deduce that such biochemical selectivity, which survived millions of years of evolution from fish to man, is one that offers the organism an adaptive advantage. While this notion has not received sufficient research attention, we note that, at least under some conditions, *RRR*-α-tocopherol maintains cell viability whereas other tocochromanols elicit cell death, e.g. desmethyl forms and especially those with delta configuration that produce highly cytotoxic aryl-quinones [27,28].

In summary, evidence that *RRR*-α-tocopherol protects against the deficiency disease in humans has been shown. Therefore, if we accept the definition of vitamin, the attribute of vitamin should be restricted to *RRR*-α-tocopherol. It is our understanding that including all tocopherols, tocotrienols and tocomonoenols in the so-called ‘vitamin E family’ is not based on direct scientific evidence and can be potentially dangerous. Indeed, the prescription of these vitamin E analogues (other than *RRR*-α-tocopherol) to protect against the deficiency disease may lead to ineffective therapy.

In this position paper we provide the scientific and medical community with what we consider relevant information in favor of revising the nomenclature of vitamin E. This suggested revision includes the following points:

1. Restrict the attribute of vitamin to *RRR*-α-tocopherol.
2. Describe only by their full chemical names other analogues, including 6-hydroxy-chromanols (i.e. non-α-tocopherols and tocotrienols) and 6-hydroxy-chromenols [16]. If a disease is identified that is specifically prevented by that compound, a tocochromanol/tocomonoenol can be correctly considered a vitamin.
3. Tocochromanols/tocomonoenols with vitamin function could be named “vitamin Ex”, where “x” indicates a number, that would be different for the different disease that is prevented. Such a nomenclature would be in analogy with the vitamins of the B group, although other means of identification could be used.

By inviting the scientific and medical communities to submit commentaries and opinions on this proposal we welcome controversial opinions, through the “Vitamin E Discussion Forum” of FRBM: https://www.editorialmanager.com/frbm/. A fair, research-based, debate will make science progress and will represent an educational example to new scientific generations.

## References

[1] Burck U, , Neuromyopathy and vitamin E deficiency in man, Neuropediatrics 12 (3) (1981) 267–278. 6945489 10.1055/s-2008-1059657

[2] Doerflinger N, , Ataxia with vitamin E deficiency: refinement of genetic localization and analysis of linkage disequilibrium by using new markers in 14 families, Am. J. Hum. Genet 56 (5) (1995) 1116–1124. 7726167 PMC1801469

[3] Ouahchi K, , Ataxia with isolated vitamin E deficiency is caused by mutations in the alpha-tocopherol transfer protein, Nat. Genet 9 (2) (1995) 141–145. 7719340 10.1038/ng0295-141

[4] Cavalier L, , Ataxia with isolated vitamin E deficiency: heterogeneity of mutations and phenotypic variability in a large number of families, Am. J. Hum. Genet 62 (2) (1998) 301–310. 9463307 10.1086/301699 PMC1376876

[5] Ulatowski LM, Manor D, Vitamin E and neurodegeneration, Neurobiol. Dis 84 (2015) 78–83. 25913028 10.1016/j.nbd.2015.04.002

[6] Traber MG, Kayden HJ, Tocopherol distribution and intracellular localization in human adipose tissue, Am. J. Clin. Nutr 46 (3) (1987) 488–495. 3630967 10.1093/ajcn/46.3.488

[7] Traber MG, , Metabolic syndrome increases dietary alpha-tocopherol requirements as assessed using urinary and plasma vitamin E catabolites: a doubleblind, crossover clinical trial, Am. J. Clin. Nutr 105 (3) (2017) 571–579. 28077381 10.3945/ajcn.116.138495 PMC5320409

[8] Nagita A, Ando M, Assessment of hepatic vitamin E status in adult patients with liver disease, Hepatology 26 (2) (1997) 392–397. 9252150 10.1002/hep.510260220

[9] Bartolini D, , Nonalcoholic fatty liver disease impairs the cytochrome P-450-dependent metabolism of alpha-tocopherol (vitamin E), J. Nutr. Biochem 47 (2017) 120–131. 28628909 10.1016/j.jnutbio.2017.06.003

[10] Violet PC, , Vitamin E sequestration by liver fat in humans, JCI Insight 5 (1) (2020). 10.1172/jci.insight.133309 PMC7030816 31821172

[11] Ran L, , Effects of antibiotics on degradation and bioavailability of different vitamin E forms in mice, Biofactors 45 (3) (2019) 450–462. 30694588 10.1002/biof.1492

[12] S M, Ataxia with vitamin E deficiency [updated 2016], in: Adam MP ED, Mirzaa GM, Pagon RA WS, Bean LJH, Gripp KW, Amemiya A (Eds.), GeneReviews^®^ [Internet], University of Washington, Seattle, Seattle (WA), USA, 2005, pp. 1993–2023. ©.

[13] Martinello F, , Supplemental therapy in isolated vitamin E deficiency improves the peripheral neuropathy and prevents the progression of ataxia, J. Neurol. Sci 156 (2) (1998) 177–179. 9588854 10.1016/s0022-510x(98)00038-0

[14] Kohlschütter A, , First recognized patient with genetic vitamin E deficiency stable after 36 Years of controlled supplement therapy, Neurodegener. Dis 20 (1) (2020) 35–38. 32623435 10.1159/000508080

[15] Gabsi S, , Effect of vitamin E supplementation in patients with ataxia with vitamin E deficiency, Eur. J. Neurol 8 (5) (2001) 477–481. 11554913 10.1046/j.1468-1331.2001.00273.x

[16] Birringer M, , N atural 6-hydroxy-chromanols and -chromenols: structural diversity, biosynthetic pathways and health implications, RSC Adv. 8 (2018) 38. 10.1039/c7ra11819h PMC9078042 35539527

[17] Azzi A, Tocopherols, tocotrienols and tocomonoenols: many similar molecules but only one vitamin E, Redox Biol. 26 (2019), 101259. 31254734 10.1016/j.redox.2019.101259 PMC6604160

[18] Traber MG, Atkinson J, Vitamin E, antioxidant and nothing more, Free Radic. Biol. Med 43 (1) (2007) 4–15. 17561088 10.1016/j.freeradbiomed.2007.03.024 PMC2040110

[19] Galli F, , Vitamin E: emerging aspects and new directions, Free Radic. Biol. Med 102 (2017) 16–36. 27816611 10.1016/j.freeradbiomed.2016.09.017

[20] Azzi A, Reflections on a century of vitamin E research: looking at the past with an eye on the future, Free Radic. Biol. Med 175 (2021) 155–160. 34478835 10.1016/j.freeradbiomed.2021.07.042

[21] Zingg JM, , In vivo regulation of gene transcription by alpha- and gammatocopherol in murine T lymphocytes, Arch. Biochem. Biophys 538 (2) (2013) 111–119. 23993952 10.1016/j.abb.2013.08.010

[22] Rota C, , Dietary vitamin E modulates differential gene expression in the rat hippocampus: potential implications for its neuroprotective properties, Nutr. Neurosci 8 (1) (2005) 21–29. 15909764 10.1080/10284150400027123

[23] Weiser H, Riss G, Kormann AW, Biodiscrimination of the eight alpha-tocopherol stereoisomers results in preferential accumulation of the four 2R forms in tissues and plasma of rats, J. Nutr 126 (10) (1996) 2539–2549. 8857515 10.1093/jn/126.10.2539

[24] Hosomi A, , Affinity for alpha-tocopherol transfer protein as a determinant of the biological activities of vitamin E analogs, FEBS Lett. 409 (1) (1997) 105–108. 9199513 10.1016/s0014-5793(97)00499-7

[25] Leonard SW, , Incorporation of deuterated RRR- or all-rac-alpha-tocopherol in plasma and tissues of alpha-tocopherol transfer protein-null mice, Am. J. Clin. Nutr 75 (3) (2002) 555–560. 11864863 10.1093/ajcn/75.3.555

[26] Traber MG, , Impaired discrimination between stereoisomers of alpha-tocopherol in patients with familial isolated vitamin E deficiency, J. Lipid Res 34 (2) (1993) 201–210. 8429255

[27] McCormick CC, Parker RS, The cytotoxicity of vitamin E is both vitamer- and cell-specific and involves a selectable trait, J. Nutr 134 (12) (2004) 3335–3342. 15570034 10.1093/jn/134.12.3335

[28] Viola V, , Mitochondrial-dependent anticancer activity of δ-tocotrienol and its synthetic derivatives in HER-2/neu overexpressing breast adenocarcinoma cells, Biofactors 39 (4) (2013) 485–493. 23361894 10.1002/biof.1089

